# Detection and Distribution of Viruses Infecting Garlic Crops in Australia

**DOI:** 10.3390/plants10051013

**Published:** 2021-05-19

**Authors:** Julia Cremer, Paul Campbell, Visnja Steele, Denis Persley, John Thomas, Stephen Harper, Cherie Gambley

**Affiliations:** 1 Department of Agriculture and Fisheries, Brisbane, QLD 4001, Australia; Julia.cremer@daf.qld.gov.au (J.C.); Paul.campbell@daf.qld.gov.au (P.C.); Visnja.steele@daf.qld.gov.au (V.S.); Denis.persley@daf.qld.gov.au (D.P.); 2 Queensland Alliance for Agriculture and Food Innovation, The University of Queensland, Brisbane, QLD 4001, Australia; j.thomas2@uq.edu.au; 3 School of Agriculture & Food Sciences, The University of Queensland, Gatton, QLD 4343, Australia; s.harper1@uq.edu.au

**Keywords:** garlic viruses, large-scale survey, molecular indexing, genetic diversity

## Abstract

The distribution of viruses in eastern Australian field garlic was evaluated. Detection assays were developed that involved generic RT-PCR for viruses in the *Allexivirus*, *Carlavirus* and *Potyvirus* genera followed by virus-specific colorimetric dot-blot hybridization. Assays targeted the potyviruses (onion yellow dwarf virus (OYDV), shallot yellow stripe virus (SYSV), and leek yellow stripe virus (LYSV)), the carlaviruses (garlic common latent virus (GCLV) and shallot latent virus (SLV)), and the allexiviruses (garlic viruses A, B, C, X (GarVA, -B, -C, -X) and shallot virus X (ShVX)). Virus incidence in crops was consistently high, with most plants infected with at least one virus from each genus. OYDV, LYSV, SLV, and GCLV were commonly detected. Three of the four allexiviruses were in all districts surveyed but varied in incidence, whereas ShVX and SYSV were not detected. There was no association between virus species complement and bulb size, indicating size is not a good predictor of the virus status of planting material. The variation of virus incidence across different Australian growing districts and in different cultivars implies multiple introductions of viruses rather than spread within the country. The genetic diversity observed within coat protein sequences of some virus species also supports multiple separate introductions.

## 1. Introduction

Garlic (*Allium sativum*, is a member of the *Amaryllidaceae* plant family and is a close relative of onion (*A*. *cepa*), shallot (*A*. *cepa* var. *aggregatum*), leek (*A*. *ampeloprasum*), and chive (*A*. *shoenoprasum*). Commercially, garlic is cultivated through vegetative propagation and is both day-length and temperature sensitive. Garlic planting windows and seasons vary considerably around Australia, and consequently a wide range of cultivars are grown. Cultivars are often locally adapted based on performance under specific day-length and temperature conditions. Australia imports around 10,000 tonnes of garlic per year, mostly sourced from China, but with increasing amounts sourced from Spain, Argentina, Mexico, and the USA. Currently, the local production of garlic in Australia is about 500 tonnes per annum, spread across different climatic zones from subtropical south-east Queensland to temperate Victoria and South Australia [1].

**Figure 1:**
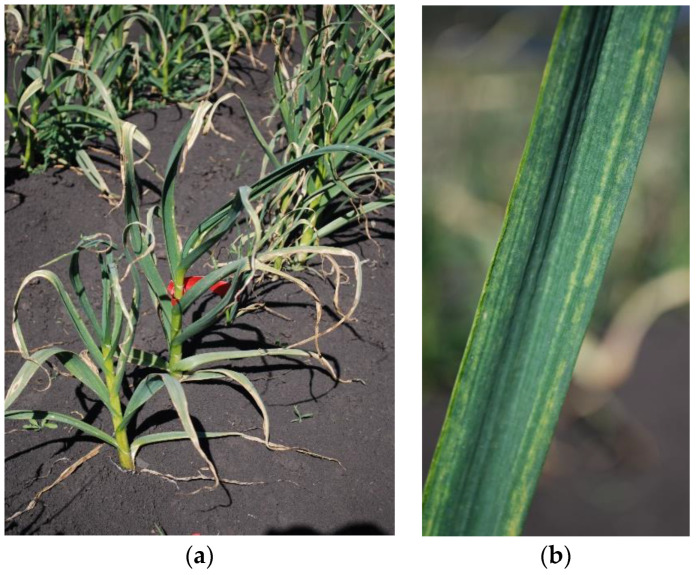
Photographs of commercial garlic affected by garlic mosaic disease showing whole field plants (**a**) and a close-up of leaf symptoms (**b**). The field plants were from survey site WP285.

Chronic virus infection of garlic is common worldwide and has a significant impact on crop quality and yield [2,3,4]. Mixed infections result in the formation of a complex, known as garlic mosaic disease, with symptoms including leaf chlorotic striping, stunted growth, and substantial reductions in bulb weight (Figure 1). Infections by different virus species, in complex, are also known to have synergistic effects that exacerbate disease symptoms and impose substantially greater limitations on plant growth and development [5,6,7]. Field bulbs are sourced as planting material for subsequent crops and are a major reservoir for viruses. Since commercial garlic production relies on this vegetative propagation, the spread of viruses is facilitated across field generations and geographical locations. Virus-free plants generated through tissue culture have shown an increase in bulb weight of 30–200% across many cultivars [8], and the use of virus-free material is demonstrated to provide substantial yield gains over several growing seasons [7]. Virus-free planting schemes for garlic, however, have not been widely adopted in Australia despite these published gains in productivity.

There are at least twelve different virus species that infect alliums. Of these, the most common are the potyviruses, *leek yellow stripe virus* (LYSV), *onion yellow dwarf virus* (OYDV), and *shallot yellow stripe virus* (SYSV) [9]; the carlaviruses, *shallot latent virus* (SLV—syn. garlic latent virus) and *garlic common latent virus* (GCLV) [10]; the allexiviruses, garlic virus A, B, C, D, E, and X (GarVA, -B, -C, -D, -E, and -X) [11,12,13]; and the tospovirus, *Iris yellow spot virus* (IYSV) [14,15]. The greatest economic losses in garlic crops have so far been attributed to the potyviruses, predominantly OYDV, and to a lesser extent, LYSV [7].

Globally, numerous studies have identified and characterized the viruses infecting garlic and established their effect on yield [5,7,9,16,17,18,19,20]. In Australia, there is scant knowledge of virus distribution and impact in commercial crops. Limited work was done in Victoria in the 1990s, with samples sourced from unidentified locations within Australia, showing the presence of OYDV, “Garlic yellow streak virus” (GYSV), and a putative unidentified carlavirus [21,22,23]. GYSV has subsequently been shown to be synonymous with LYSV [24]. Additionally, *lettuce necrotic yellows virus* (LNYV) was reported from diseased field garlic from central Victoria [21]. The presence of allexiviruses (GarVA, GarV-B, GarV-C, GarV-D, and GarV-X), carlaviruses (GCLV and SLV), and potyviruses (OYDV and LYSV) were reported in garlic from Western Australia [25,26]. These studies, however, were limited to only a few samples of imported retail garlic bulbs, one retail nursery plant, one locally-grown home garden plant, and one local organic commercial field plant. The current virus status of commercial Australian garlic crops is unknown and warrants further study. Given the likely multiple introductions of garlic germplasm into Australia to suit the different climatic production zones, it is possible the distribution of virus species would vary across these zones. Additionally, genetic variation within some virus species affecting garlic is known to be divergent [25].

The aim of this study was to determine the identity and distribution of viruses in eastern Australian commercial garlic crops. To efficiently assess disease survey samples for viruses in the genera *Potyvirus*, *Carlavirus*, and *Allexivirus*, a novel molecular diagnostic method was developed. The new method aimed to detect all 10 target viruses using RT-PCR with a single reverse transcriptase reaction, primed with an oligo-dT and then three genus-specific forward primers in PCR. Individual virus species were then identified from the RT-PCR amplicons with specific probes using a dot blot hybridization assay. The distribution of virus species within planting propagules was also examined to investigate the hypothesis that all cloves from an infected bulb are uniformly infected.

## 2. Results

### 2.1. Molecular Detection of Garlic Viruses

The method developed in this study allowed efficient indexing of over 3500 field plant samples and was used to evaluate trial plants. The genus-level degenerate RT-PCR successfully amplified the expected viruses, and individual probes identified the specific viruses. The results from up to 100 selected samples per virus were verified using specific RT-PCR for GarVA, GarVB, GarVC, GarVX, GCLV, SLV, OYDV, and LYSV and confirmed the accuracy of the probe-based assay.

Indexing for IYSV was done separately using a previously published assay [27], as it is the only virus within the Tospovirus genus likely to occur in commercial Australian garlic.

### 2.2. Field Surveys

This work represents a large-scale evaluation of virus distribution across several of Australia’s main garlic producing regions (Table 1). The genus-level degenerate RT-PCR provided an overall view of the infection status of the surveyed crops, and profiling of species of individual viruses infecting the surveyed plants was achieved through hybridization assays with labelled probes specific to 10 viruses known to infect garlic (Table 1).

Virus incidence detected by genus-specific RT-PCR within the surveyed crops ranged from 90 to 100% across individual samples from Queensland sites (Table 1). Incidences estimated from the bulked samples collected from other States were similar, although there was more variation for allexivirus and potyvirus incidence, than for carlaviruses. In most cases, mixed infections were detected, with virus from at least one species from each genus present in the plant, though the profile of viruses from individual species infecting each crop varied across sites.

**Figure 2:**
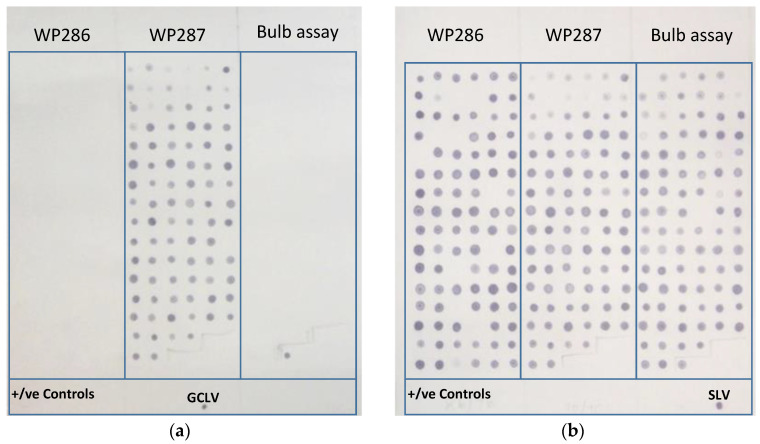
An example of probe hybridization-based detection of the carlaviruses (**a**) garlic common latent virus (GCLV) and (**b**) shallot latent virus (SLV). Garlic plant samples were from two survey sites in Queensland, Lower Tenthill (WP286), and Kalbar (WP287) and from the plant propagation experiment (bulb assay) carried out under glasshouse conditions in Queensland. Each panel of the membrane represents a 96-well plate of bulked samples from each property, with all positive controls (plasmid) blotted across the bottom of the membrane.

Species-specific virus testing showed that plants were chronically infected with the potyvirus OYDV, the carlavirus SLV, and the allexiviruses GarVA, GarV-B, and GarV-X (Table 1). High incidences of SLV and OYDV were detected throughout crops in every growing district surveyed (Figure 2, Table 1). LYSV infections appeared to be fairly widespread, reaching similar levels to OYDV in Queensland crops, where the virus was found to be present in 77.7–100% of plants surveyed from the different sites. However, the virus was less common in Victoria, South Australia, and New South Wales, where LYSV infected less than 25% of the plants surveyed. SYSV was not identified in the current study across any of the surveyed areas. The distribution of the carlavirus GCLV was variable, with high levels at half of the Queensland and about one-third of the Victorian properties surveyed (Table 1). For example, the incidence of GCLV in crops at Mt. Sylvia (WP288) and Kalbar (WP287) was approximately 90% or more, while the virus was not detected at the site at Lower Tenthill (WP286) (Figure 2, Table 1). The results for this site (WP286) also indicate the possibility of a third, as yet unknown, carlavirus present in Australian garlic. At WP286, 93/94 samples were positive for carlavirus, but SLV was only identified in 84 samples and GCLV not at all. This indicated there were nine plants that were positive using the generic assay, for which there was no virus detected using the specific assays. No further testing of this material was completed; the possibility of the presence of a third carlavirus requires further investigation.

Profiles of the allexiviruses varied distinctly across growing districts. Crops tested from districts in South Australia and New South Wales displayed lower virus incidences than those from Queensland and Victoria, particularly at Renmark, South Australia, where only very low levels (<8% of GarVX) were detected. In Queensland, GarVA, -B, and -X were present at high levels, >50%, throughout most crops. In Victoria, GarVX was more commonly detected than GarVA and GarVB. GarVA was generally present in the Queensland crops tested, while in material surveyed from southern Australia, the virus was only detected on two Victorian properties (Table 1). These included East Gippsland, Victoria (Site 1), where high levels (up to 100%) of GarVA were detected, and Daylesford, Victoria (Site 8), where the virus was detected in <5% of plants tested. GarVC incidences were low to negligible across all Australian districts surveyed. ShVX was also not identified in the current study across any of the surveyed areas.

Only the Queensland surveyed crops were tested for the tospovirus IYSV, and it was not detected in any samples.

### 2.3. Evaluation of Planting Propagules for Virus Distribution

The incidence of viruses of individual species within cloves of field grown bulbs was evaluated through indexing plantlets derived from viable cloves. A comparison of virus incidences was made between planting material selected as either large bulbs (>80 g) or small bulbs (20–30 g) (Table 2). The average weight of the seven large bulbs evaluated was 86.6 g. These had an average of 16.1 cloves/bulb at 6 g/clove. By contrast, the average weight of the small bulbs was 24.7 g, with 7.2 cloves/bulb and an average clove weight of only 3.3 g. Consequently, 14 small bulbs were required to evaluate a similar number of total cloves as those from the large bulbs. For large bulb material, 110 individual viable cloves were assessed, and for small material, 92. The emergence rate of cloves from all bulbs was 93–100%. All plantlets showed leaf mosaic/streaking symptoms, and the severity of the symptoms was similar, irrespective of the size rating of original bulb material.

Virus incidence within the clove material was very high, with all six viruses detected (GarVA, GarVB, GarVX, SLV, OYDV, and LYSV; Table 2) present in approximately 87 and 77% of individual cloves from large and small bulbs, respectively. The suite of virus species infecting cloves from individual bulbs didn’t vary greatly for the large quality bulbs, with incidences for individual viruses ranging from 80–100% (Table 2). By contrast, the suite of virus species infecting cloves from small quality bulbs did vary, and individual virus incidences ranged from as low as 25% of cloves from a single bulb to 100% (Table 2). In this material, the majority of variability was due to the absence of one or more of the allexiviruses. Multivariate analysis of the weights and virus infection status (average % cloves infected) of small and large quality bulbs did not show a positive correlation between yield and virus infection status.

### 2.4. Genetic Diversity and Phylogenetic Analysis of Viruses Present in Australian Garlic Crops

Nucleotide alignments of coat protein (CP) sequences for the selected viruses detected in the surveyed Australian garlic were compared with isolates from GenBank.

**Figure 3:**
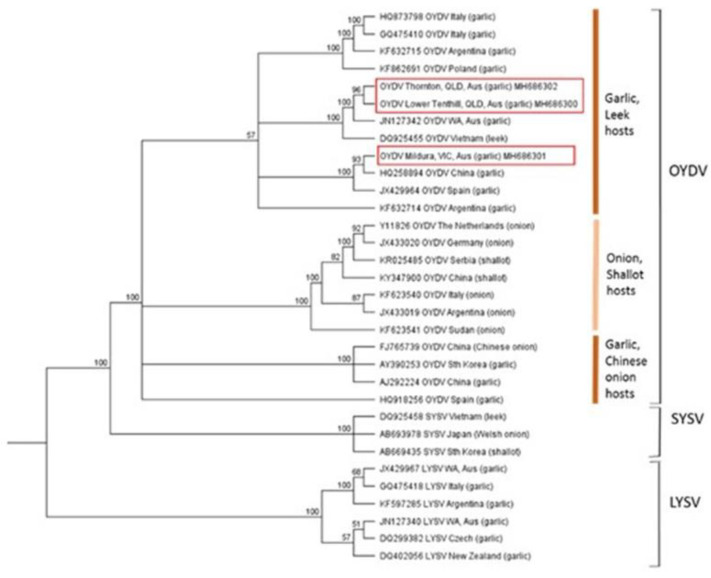
Phylogenetic tree of potyviruses generated from a multiple nucleotide sequence alignment of the coat protein coding region using the Geneious tree builder function, with the genetic distance model Tamura-Nei and the UPGMA tree build method. A bootstrap resampling method with 100 replicates was applied to generate a consensus tree with a support threshold of 50%. Australian isolates are outlined in red boxes, and host groupings for onion yellow dwarf virus (OYDV) are shown with vertical lines and host labels.

The potyviruses clustered in three well-supported clades, representing OYDV, SYSV, and LYSV (Figure 3). OYDV CP nucleotide sequences included in the analyses were 80.1–99.7% identical (Table S5), which is within the species demarcation limit for potyvirus species [28]. The two OYDV isolates from Queensland clustered most closely with an isolate from Western Australian garlic (98.8% identity, GenBank JN127342) and a leek isolate from Vietnam (93.3% identity, GenBank DQ925455). The Victorian garlic isolate clustered separately and was closest to a Chinese isolate (GenBank HQ258894).

**Figure 4:**
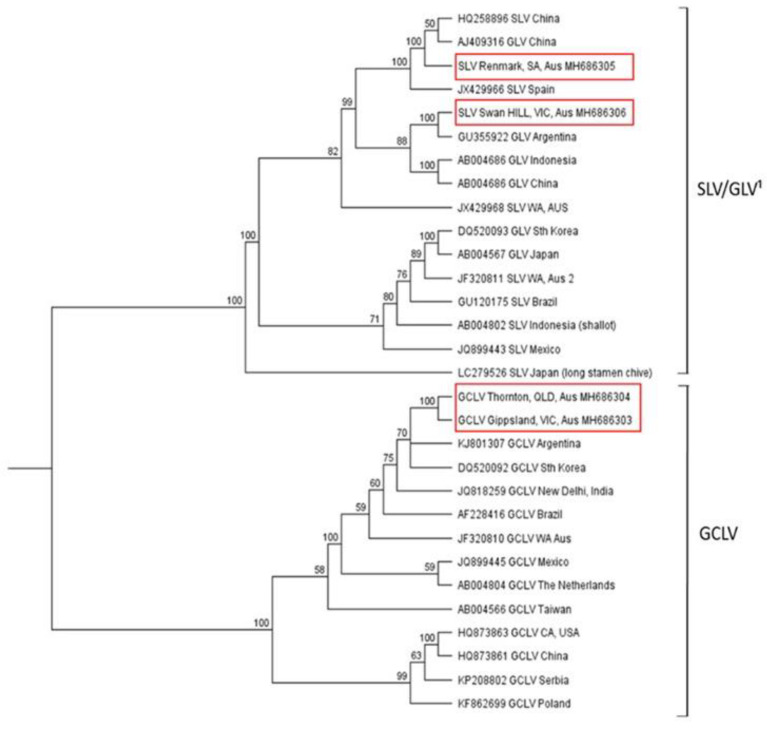
Phylogenetic tree of carlaviruses generated from a multiple nucleotide sequence alignment of the coat protein coding region using the Geneious tree builder function, with the genetic distance model Tamura-Nei and the UPGMA tree build method. A bootstrap resampling method with 100 replicates was applied to generate a consensus tree with a support threshold of 50%. Australian isolates are outlined in red boxes.

For the carlavirus sets, GCLV CP nucleotide sequences were 88–99.6% identical, and for SLV they were 78.1–99% identical (Figure 4, Table S6). The GCLV isolates from this study were genetically very similar (99.2% identity), despite their collection from the distantly located growing regions of Thornton, Queensland, and Gippsland, Victoria. The closest sequences to these Australian GCLV isolates was an isolate from South Korea, which was 96.5–96.8% identical to the Australian isolates, whereas the sequences were only 94% identical to the isolate reported from Western Australia (GenBank JF320810). In contrast, SLV nucleotide sequence variation indicated that the Australian isolates detected in this study were more closely related to overseas isolates than to each other. They shared an identity of 92.6%, whereas the isolate from Swan Hill, Victoria, was most closely related to an Argentinian isolate (97.5% identity), and the isolate from Renmark, SA, aligned most closely with one from China (98.9% identity).

**Figure 5:**
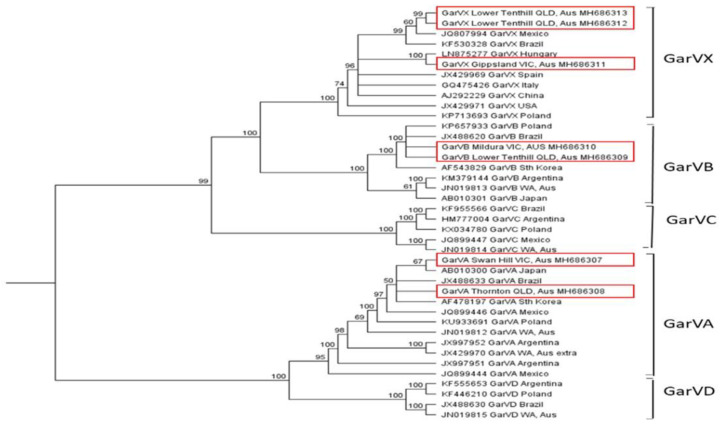
Phylogenetic tree of allexiviruses generated from a multiple nucleotide sequence alignment of the coat protein coding region using the Geneious tree builder function, with the genetic distance model Tamura-Nei and the UPGMA tree build method. A bootstrap resampling method with 100 replicates was applied to generate a consensus tree with a support threshold of 50%. Australian isolates are outlined in red boxes.

The allexiviruses formed two well-supported clades on the phylogenetic tree, with one containing GarVA and GarVD, and the other GarVB, GarVC, and GarVX (Figure 4). Nucleotide sequence identities of GarVA exhibited a high level of diversity, with some isolates sharing only 71.6% identity, while minimum intraspecies identities were 88% for GarVB and 91.3% for GarVX (Table S7). Across growing districts, GarVA isolates from Queensland and Victoria were closely related, with 99.4% identity. Furthermore, several GarVA isolates from overseas, including isolates from Japan, Brazil, and Korea, also clustered closely with the Australian isolates and exhibited identities of 98.8–99.5% (Figure 5, Table S7). GarVB showed a similarly high level of identity among isolates from Queensland and Victoria at 98.1%, with the closest overseas isolates having 98.2% identity to these Australian isolates. High sequence identity (99.5%) was also observed between two GarVX isolates from Lower Tenthill, Queensland, however, they grouped more distantly to the Victorian isolate (94.5%), reflecting a greater diversity across growing districts. The sequences of GarVA and GarVB from the current study were 84.6–85.1% and 88–89% identical, respectively, to isolates of these viruses previously reported from Western Australia (GenBank JN019812 and JN019813) [25].

## 3. Discussion

This study presents the first comprehensive virus survey of Australian garlic crops. Surveying of a range of garlic cultivars from plantings across four Australian States ensured a widespread assessment of the virus status of crops comprising a range of commercial cultivars across the industry. Symptoms of virus infection were present in all plants, and virus presence was confirmed by molecular diagnostic testing. Virus-free planting material was distributed to some Victorian growers in the 1990s [22] and may have contributed to the observed variation in virus distribution in growing regions in this state; however, information on which growers received this material is no longer available.

Molecular testing showed that Australian field-grown garlic cultivars are chronically infected with a complex of viruses, with more than 90% of plants infected with viruses from one or more species from each of the three genera tested. No single infections were recorded. Probe hybridization assays showed that the potyvirus OYDV and the carlavirus SLV were the most widespread viruses found in the surveyed crops. This result contrasts starkly with that of Sward [22], who failed to detect SLV in any samples. LYSV was present at comparable incidences to OYDV in Queensland crops but was not as widespread in growing districts of other States, where less than 25% of surveyed plants were infected. GCLV and a varied profile of the allexiviruses GarVA, GarVB, and GaVX were also commonly detected. GCLV is not uniformly present in Australian garlic crops. In the current study, infection rates were more variable than for either SLV or OYDV, with individual crops in Queensland exhibiting 0–100% incidence. GCLV was not detected at surveyed sites in Renmark, South Australia, whereas some crops in Victoria were heavily infected (up to 100% incidence), and several crops in Victoria and one in New South Wales showed moderate levels of GCLV (~20% incidence). This work appears to provide the first field detection of GarVB, GarVC, and GarVX in Australian commercial garlic crops.

Although a limited number of samples were examined for virus diversity, phylogenetic analysis of partial or complete CP sequences from this work and that of Wylie [25] revealed genetically diverse isolates of OYDV, LYSV, GCLV, SLV, GarVA, GarVB, and GarVX. In many cases, these virus isolates also displayed a high degree of identity to overseas isolates. These data indicate that multiple introductions of garlic viruses into Australia have occurred, and that the cultivars grown in Australia contain a varied complement of viruses and virus strains.

The allexivirus ShVX and the potyvirus SYSV were not reported in the current study of surveyed field garlic nor in any of the previous studies, so it is unlikely that these viruses are present in Australian garlic. IYSV, a tospovirus, is known to infect alliums such as onions, chives, leeks, and garlic, and in Australia it has been recorded from onion crops in WA, NSW, and Victoria [27]. In the current study, IYSV was not detected by specific RT-PCR in Queensland garlic crops despite its previous detection in onion and shallot crops within the same production district (D. Persley, unpublished). IYSV is regularly recorded in Australia in onion crops grown from true seed [27].

The evaluation of virus distribution within large and small bulb planting material showed that none of the plantlets that emerged from the cloves were virus free, and almost all were found to have mixed infections, predominantly comprising OYDV, LYSV, SLV, and one or more of the three allexiviruses: GarVA, GarVB, and GarVX. Plantlets derived from cloves from large bulbs were almost uniformly infected with all six viruses. By contrast, the virus incidence in plants derived from cloves from small bulbs was variable. In most instances where there were non-uniform virus infections, it was due to an absence of one or more of the allexiviruses. No correlation was observed between reduced clove size and virus complement. Instead, clove size varied independently of infection status in this experiment.

These results highlight two key aspects of garlic virus disease. First, infection of garlic with viruses from multiple species per se doesn’t necessarily correlate well with yield reductions, as the bulbs in this study were all from a single cultivar sourced from one property and were infected with the same range of virus species, irrespective of their size. Thus, the selection of planting material based on size or quality is unlikely to significantly reduce virus incidence in subsequent crops. However, the titer of individual viruses may be an important factor in determining the impact of viruses on yield as opposed to simply the presence of the virus, and selective gene silencing may play a role. The second point is that bulb size is not a good indicator of virus status for use as planting material, as cloves from the smaller bulbs sometimes contained fewer viruses than cloves from the larger bulbs. However, bulb size is a good indicator of yield in the subsequent crop, and research has shown that the consistent re-selection of the largest bulbs with more vigorous blue-green foliage (albeit still with virus symptoms) provides substantial generational yield improvement (S. Harper, unpublished).

The high incidence of viruses in garlic cultivars across all Australian growing areas and the recorded yield benefits of growing virus-free garlic [2,3,4,5,7] suggest value in pursuing the production of virus-free garlic for the Australian industry.

## 4. Conclusions

This study evaluated the distribution of viruses in eastern Australian field garlic and provided new methodology for multiplex detection of 10 individual virus species. The variable distribution of individual virus species across the different growing districts and cultivars implies multiple introductions of viruses into Australia rather than spread within the country. The genetic diversity observed within coat protein sequences of some virus species also supports multiple independent introductions. Evaluation of planting propagules indicated bulb size was not a good predictor of the virus status of planting material; however, it has potential as a predictor of subsequent yield.

## 5. Materials and Methods

### 5.1. Virus Indexing

To efficiently test for the 10 virus species, molecular indexing involved RT-PCR amplification using genus-specific primers for allexiviruses, carlaviruses, and potyviruses. This was coupled with species-specific dot blot hybridization assays (DIG-Easy Assay, Roche, North Ryde NSW, Australia) to identify the individual virus species. IYSV was tested using specific primers for the virus, as described by [29]. Details of the samples used to generate species-specific probes are listed in Table S1. Information pertaining to the primer sequences, annealing temperature, and product size for each primer combination is listed in Table S2.

Total nucleic acid was extracted from leaf tissue samples using the Biosprint DNA Plant Kit, according to the manufacturer’s instructions (Qiagen, Hilden, Germany), though omitting the RNAse from the buffer RPW. The synthesis of cDNA was carried out using the reverse transcription enzyme Superscript III (Thermofisher Scientific, Waltham, MA USA) and the universal Poty1 reverse primer (Table S2) according to the manufacturer’s protocol. This primer was used for cDNA synthesis, as all target viruses are polyadenylated. The cDNA was amplified in PCR using a combination of degenerate forward primers specific for each virus genus and the reverse primer Poty1.

Genus level PCRs contained 10 pmol degenerate forward primer corresponding to the virus genus being amplified (Alcar1, U341 and PGVt3, Table S2), 10 pmol reverse primer Poty1, 1.75 mM MgCl_2_, 100 µM dNTPs, 2.0 µL cDNA, and 0.2 U Taq Polymerase (Thermofisher Scientific, Waltham, MA USA) in a total volume of 25 µL. The PCR cycle parameters were as follows: initial denaturation 94 °C for 1 min, then 35 cycles of 94 °C for 20 s, 56 °C annealing for 60 s, 72 °C extension for 60 s, and a final extension at 72 °C for 3 min. For IYSV, PCR cycle conditions were as follows: 94 °C for 2 min, 35 cycles of 94 °C for 30 s, 48 °C for 30 s, and 72 °C for 45 s, followed by a final extension at 72 °C for 5 min.

For dot-blot hybridization assays, probes were generated through PCR amplification of approximately 1 ng/µL of recombinant plasmid from selected virus isolates sourced from Australia or introduced under permit from overseas (Table S1). Specific probes were prepared for GarVA, -B, -C, and -X, ShVX, GCLV, SLV, LYSV, OYDV, and SYSV. Reference isolates were not available for GarVD and GarVE, so it was not possible to develop probes for these viruses, and thus they were not surveyed in this study. The recombinant plasmids were prepared by amplification of the 3′-end of viral genomes using the genus level primers as described above and subsequent insertion of the amplicons into TOPO plasmid vectors using the TA cloning kit (Invitrogen). Identity of the cloned virus inserts was confirmed by Sanger sequencing. Probe labelling PCR used 5 pmol species-specific primers (Table S3), 1 ng recombinant plasmid, 100 µM digoxigenin-d-UTP labelled nucleotides (Roche), 0.875 µL MgCl_2_, 0.5 µL dNTPs, 2.6 µL d-UTP, and 0.3 µL Mango Taq polymerase (Bioline). The cycling conditions included an initial denaturation at 95 °C for 1 min, followed by 25 cycles of 95 °C for 15 s, 56 °C for 20 s, 72 °C for 40 s, and a final extension step of 72 °C for 5 min. For GarVC, the annealing temperature was adjusted to 50 °C.

To prepare dot blots, 3 µL of generic PCR product from each sample was added to 27 µL of denaturation solution (12 µL of 1 M NaOH, 3 µL of 0.1 M EDTA, and 12 µL dH_2_O) and applied to Hybond-N+ membrane (Roche) in 1 µL volumes using a grid pattern (Figure 2). A separate blot was prepared for each of the 10 viruses tested, and each membrane contained the positive control aliquots for all 10 viruses, added as approximately 1 µg of recombinant plasmid. The membranes were pre-hybridized by incubation with DIG-easy Hyb solution (20 ml/100 cm^2^) (Roche) at 42 °C for 30 min with rotation. Excess hyb-solution was removed, and the denatured virus-specific digoxigenin-labelled probe was added. Prior to hybridization, probes were first denatured by incubation at 95 °C for 5 min, then placed onto ice. The probes were then diluted in DIG-Easy hyb at a rate of 3.5 mL of diluted probe per 100 cm^2^ of membrane, added to the membrane, and incubated overnight at 42 °C with rotation.

To remove the unbound probe, membranes were washed with 2 x SSC, 1% SDS at room temperature for 15 min, followed by a higher stringency wash with 0.1 x SSC, 1% SDS at 68 °C for 15 min. To visualize the bound probe, membranes were developed using an anti-DIG alkaline phosphatase (AP) conjugate with an AP conjugate color substrate (AP color reagent A + B, Biorad) following the DIG-detection kit, as per the manufacturer’s instructions. The color substrate was left to develop for at least 30 min. The reaction was then stopped by washing membranes in distilled water.

### 5.2. Field Surveys

Field surveys of Queensland garlic crops from four properties (Table 1) were completed in 2013, where it was found that all inspected plants displayed typical symptoms of virus infection. Approximately 100 plants were sampled randomly from each block and processed individually, except for site WP285, where 238 samples were collected. In 2015, additional field surveys were conducted on a selection of garlic crops from eight sites in Victoria, one site in South Australia, and one in New South Wales (Table 1). Similarly, virus symptoms were obvious on all plants inspected in all crops. Random samples were collected from each of the 2015 survey sites, and 300 individual leaves per site were bulked in 30 batches of ten for molecular indexing. The incidence of virus in bulked samples was determined as described by Moran et al. [30] and reported as estimated incidence plus 95% upper and lower confidence intervals.

### 5.3. Evaluation of Planting Propagules for Virus Distribution

A pot trial was done to investigate the distribution of individual virus species in planting propagules (i.e., cloves) from infected bulbs. Bulbs were selected from symptomatic plants of cultivar Glenlarge from a single Queensland property in 2013 and separated based on weight into large (>80 g) and small (20–30 g) bulbs. Cloves from all test bulbs were planted individually and grown in a temperature-controlled glasshouse environment. To generate approximately 100 cloves per size class for evaluation, 14 bulbs of the small size were used, whereas only seven of the larger bulbs were required. The quality of the planting material was evaluated for viability, average clove weight, and number of cloves per bulb. A total of 92 cloves produced plantlets from small sized bulbs, and 110 from the larger bulbs. The subsequent plantlets were maintained insect- and mite-free to prevent cross-infection of viruses, rated for virus symptoms and indexed for individual virus species using RT-PCR dot blot hybridization described above. Principal component analysis (PCA) in the statistical software package Minitab was employed to measure correlation between the weight (grams) of large- and small-sized bulbs and their virus infection status and average % cloves infected with individual viruses within the bulb.

### 5.4. Phylogenetic Analysis of the Virus Coat Protein Gene

Direct sequencing of the coat protein (CP) coding region was completed for several representative survey samples for the various garlic viruses identified. Using Geneious software, primers were designed to amplify the partial or complete CP-encoding region of the identified viruses. The CP sequencing involved PCR amplification of 2 µL cDNA (described above) with 10 pmol targeted primers in a 25 µL reaction containing 0.3 µL Taq polymerase (Mango Taq, Bioline, Eveleigh NSW, Australia). PCR cycling conditions were as follows: initial denaturation 95 °C for 1 min, followed by 35 cycles of 95 °C for 15 s, 48–62 °C for 20 s, 72 °C for 60 s, and a final extension of 72 °C for 5 min. See Supplementary data 1b for CP primer sequences, product sizes, and annealing temperatures for each virus. PCR products were visualized on a 1.5% agarose gel stained with ethidium bromide. PCR reactions were prepared for direct sequencing by enzyme treatment. For this, 2 µL of a mix containing 1 U/µL Exonuclease I and 1 U/µL Antarctic Phosphatase (ExoAP) (New England Biolabs, Notting Hill, VIC, Australia) was added to each 20 µL PCR reaction and heated to 37 °C for 20 min, which was then followed by incubation at 80 °C for 10 min to inactivate the enzymes. Some PCR amplicons required gel purification using the PCR gel extraction kit (Bioline). Purified PCR amplicons were sequenced in both directions by Macrogen (Geumcheon-gu, Seoul, South Korea) (dna.macrogen.com) using Sanger sequencing methods. Sequences were edited to remove primer sequences, and a consensus sequence for each PCR amplicon was generated in Geneious. The nucleotide sequences were aligned and compared to related isolates sourced from GenBank. All sequences included in the alignment were trimmed to only contain the CP region of the genome.

Using the Geneious software, a global multiple sequence alignment was created, and phylogenetic trees were generated using genetic distance model Tamura-Nei and the UPGMA tree build method setting. Distances were obtained from pairwise alignments of all sequences. A bootstrap resampling method with 100 replicates was applied to generate a consensus tree with a support threshold of 50%.

## Figures and Tables

**Table 1:** Results for virus detection in Australian surveyed garlic. From each survey, plant samples were tested individually for sites WP285-288. For the remaining sites, 300 individual samples were bulked in groups of 30 samples, each containing tissue from 10 individuals. The samples were tested for the three different virus genera (allexivirus, carlavirus, and potyvirus) by degenerate RT-PCR, then for the presence of individual virus species, through hybridization assay. Virus acronyms are as follows: GarVA, -B, -C, and -X = garlic virus A, -B, -C, and -X; GCLV = garlic common latent virus; SLV = shallot latent virus; OYDV = onion yellow dwarf virus; SYSV = shallot yellow stripe virus; LYSV = leek yellow stripe virus. The results are reported as a percentage incidence, with a range provided for bulked samples.

Survey Site	Location	Variety	Number of Samples	Allexiviruses	GarVA	GarVB	GarVC	GarVX	ShVX	Carlaviruses	GCLV	SLV	Potyviruses	OYDV	SYSV	LYSV
WP285	Thornton, QLD	Glenlarge	238	100.0	66.4	62.6	2.9	80.3	0.0	99.6	2.9	100.0	99.6	97.9	0.0	97.5
WP286	Lower Tent Hill, QLD	Glenlarge	94	98.9	65.6	93.8	30.2	62.5	0.0	98.9	0.0	89.6	98.9	94.8	0.0	87.5
WP287	Kalbar, QLD	Glenlarge	90	99.0	52.2	91.1	0.0	30.0	0.0	100.0	98.9	100.0	91.1	98.9	0.0	100.0
WP288	Mt Sylvia, QLD	Glenlarge	94	94.0	84.0	92.6	3.2	61.7	0.0	98.9	89.4	80.9	91.5	67	0.0	77.7
Site 1	East Gippsland, VIC	Purple stripe	30	100.0 (19.4–100)	100.0 (19.4–100)	0.0 (0–1.2)	0.7 (0.08–1.9)	28.8 (16.1–51)	0.0 (0–1.2)	100.0 (19.4–100)	13.5 (8.2–20.6)	100.0 (19.4–100)	20.6 (12.4–30.0)	20.6 (12.4–32)	0.0 (0–1.2)	4.0 (1.9–7.2)
Site 2	East Gippsland, VIC	not known	30	23.7 (14.0–38.1)	0.0 (0–1.2)	20.6 (12.4–32)	2.6 (1.04–5.3)	0.0 (0–1.2)	0.0 (0–1.2)	23.7 (14.0–38.1)	11.3 (6.8–17.4)	16.4 (10–25)	100.0 (19.4–100)	100.0 (19.4–100)	0 (0–1.2)	14.9 (9.1–22.6)
Site 3	Swan Hill, VIC	Chinese white	30	100.0 (19.4–100)	0.0 (0–1.2)	14.9 (9.1–22.6)	0.7 (0.1–1.9)	100.0 (19.4–100)	0.0 (0–1.2)	100.0 (19.4–100)	100.0 (19.4–100)	100.0 (19.4–100)	100.0 (19.4–100)	100.0 (19.4–100)	0.0 (0–1.2)	3.8 (5.1–13.8)
Site 4	Merbein, VIC	Red	30	100.0 (19.4–100)	0.0 (0–1.2)	18.2 (11.1–28)	1.05 (0.2–3.03)	100.0 (19.4–100)	0.0 (0–1.2)	100.0 (19.4–100)	100.0 (19.4–100)	100.0 (19.4–100)	28.8 (16.1–51.0)	16.4 (10–25)	0.0 (0–1.2)	0.0 (0–1.2)
Site 5	Merbein, VIC	not known	30	28.8 (16.1–51.0)	0.0 (0–1.2)	20.6 (12.4–32)	0.7 (0.08–1.9)	28.8 (16.1–51)	0.0 (0–1.2)	100.0 (19.4–100)	3.5 (1.6–6.6)	100.0 (19.4–100)	28.8 (16.1–51.0)	23.7 (14–38.1)	0.0 (0–1.2)	4.0 (1.9–7.2)
Site 6	Mildura, VIC	Red	30	16.4 (10.0–25.0)	0.0 (0–1.2)	14.9 (9.1–22.6)	0.3 (0.01–1.9)	13.5 (8.2–20.6)	0.0 (0–1.2)	100.0 (19.4–100)	28.8 (16.1–51)	100.0 (19.4–100)	100.0 (19.4–100)	28.8 (16.1–51)	0.0 (0–1.2)	5.0 (2.5–8.6)
Site 7	Mildura, VIC	not known	30	28.8 (16.1–51.0)	0.0 (0–1.2)	20.6 (12.4–32)	0.0 (0–1.2)	28.8 (16.1–51)	0.0 (0–1.2)	100.0 (19.4–100)	100.0 (19.4–100)	100.0 (19.4–100)	28.8 (16.1–51.0)	28.8 (16.1–51)	0.0 (0–1.2)	2.2 (0.8–4.8)
Site 8	Daylesford, VIC	Purple stripe	30	28.8 (16.1–51.0)	2.0 (0.8–4.8)	0.0 (0–1.2)	14.9 (9.1–22.6)	18.2 (11.1–28)	0.0 (0–1.2)	100.0 (19.4–100)	3.1 (1.3–6)	100.0 (19.4–100)	100.0 (19.4–100)	28.8 (16.1–51)	0.0 (0–1.2)	8.2 (11.1–28)
Site 9	Buronga, NSW	Various	30	100.0 (19.4–100)	0.0 (0–1.2)	6.4 (10–25)	0.0 (0–1.2)	0.3 (0.01–1.9)	0.0 (0–1.2)	100.0 (19.4–100)	18.2 (11.1–28)	100.0 (19.4–100)	100.0 (19.4–100)	100.0 (19.4–100)	0.0 (0–1.2)	3.5 (1.6–6.6)
Site 10	Renmark, SA	Purple	30	5.0 (2.5–8.6)	0.0 (0–1.2)	0.0 (0–1.2)	0.0 (0–1.2)	4.45 (2.2–7.9)	0.0 (0–1.2)	100.0 (19.4–100)	0.0 (0–1.2)	100.0 (19.4–100)	100.0 (19.4–100)	28.8 (16.1–51)	0.0 (0–1.2)	1.8 (0.6–4.2)

**Table 2:** Virus distribution within large- and small-sized bulbs. Virus incidence was evaluated by testing plantlets derived from each clove of the bulbs and used virus-specific probe hybridization of the degenerate RT-PCR assay amplicons. Virus incidence as a percentage of total cloves per bulb tested is listed, along with the total number cloves evaluated per bulb. Virus acronyms are as follows: GarVA, -B, -C, and -X = garlic virus A, -B, -C, and -X; GCLV = garlic common latent virus; SLV = shallot latent virus; OYDV = onion yellow dwarf virus; LYSV = leek yellow stripe virus.

Bulb Quality	Bulb Reference	Number of Cloves Evaluated	GarVA	GarVB	GarVX	SLV	OYDV	LYSV
Small	1	9	71	86	71	86	86	86
	2	10	100	70	70	90	90	80
	3	4	100	100	100	100	100	100
	4	6	100	100	100	100	100	100
	5	4	100	100	100	100	100	100
	6	12	100	83	100	92	83	83
	7	10	90	100	100	100	100	100
	8	3	100	100	100	100	100	100
	9	4	100	100	100	100	100	100
	10	11	64	73	73	100	100	100
	11	4	50	25	75	75	100	100
	12	6	67	67	83	100	100	83
	13	4	100	100	100	100	100	100
	14	5	60	60	60	80	100	100
Large	1	17	100	100	82	100	100	100
	2	15	100	100	93	100	93	100
	3	16	100	100	100	100	100	100
	4	18	94	94	94	94	100	94
	5	12	92	92	92	92	100	92
	6	15	100	100	80	87	100	100
	7	17	94	94	94	94	100	100

## Data Availability

Australian virus coat protein nucleotide sequences generated in the study were deposited in GenBank under accession numbers MH686300-313.

## Supplementary Materials

The following are available online at https://www.mdpi.com/article/10.3390/plants10051013/s1, Table S1 Record of reference isolates, their host species and geographic origin, used to generate probes through specific RT-PCR; Table S2, Primer sequence information for amplification of degenerate PCR products for the allexi-, carla-, and potyviruses; Table S3, Primer sequence information for PCR labelling of species-specific probes; Table S4, Primer sequence information for amplicon sequencing of the coat protein (CP) of nine garlic virus species; Table S5, Distance matrix showing percent nucleotide identities of coat protein sequences of potyviruses; Table S6, Distance matrix showing percent nucleotide identities of coat protein sequences of carlaviruses; Table S7, Distance matrix showing percent nucleotide identities of coat protein sequences of allexiviruses.
