# Supplementary material for: Detection and Distribution of Viruses Infecting Garlic Crops in Australia
Source: Plants (Basel). 2021 May 19;10(5):1013. doi: 10.3390/plants10051013 (PMC8160985; doi:10.3390/plants10051013)
Supplement: Supplementary file 1 [file plants-10-01013-s001.zip › plants-1197031-supplementary.pdf]

Table S1. Record of reference isolates, their host species and geographic origin, used to generate probes through specific RT-PCR. Virus acronyms are as follows: GarVA, -B, -C, and -X = *garlic virus A, -B,-C*, and *-X*, GCLV = *garlic common latent virus*, SLV = *shallot latent virus*), OYDV = *onion yellow dwarf virus*, SYSV = *shallot yellow stripe virus* and LYSV = *leek yellow stripe virus*.

Isolate number	Host species	Country of Origin	Virus specific probes
3185	*Allium sativum*, garlic	Gatton, Australia	GarVA, GarVX, GCLV, OYDV
3188	*Allium sativum*, garlic	Gatton, Australia	GarVC
3191	*Allium sativum*, garlic	Gatton, Australia	GarVB, SLV
Q4608	*Allium cepa*, shallot	Cirebon, Indonesia	SLV, SYSV
Q4609	*Allium cepa*, shallot	Indonesia (IVEGRI)	SLV
PC-0451 DSMZ	*Allium ampeloprasum* var. *porrum,* leek	Indonesia	LYSV
PV-0622	*Allium ascalonicum,* shallot	Slovenia	ShVX

Table S2 Primer sequence information for amplification of degenerate PCR products for the allexi-, carla-, and potyviruses.

Primer	Sequence (5′-3′)	Target	Product size w/ Poty1 (bp)	Reference	Use
Poty1	GGATCCCGGGTTTTTTTTTTTTTTTTTV	Poly A tail	na	Gibbs and MacKenzie, 1997	cDNA and reverse
AlcarF	TGCTGCYTTTGATACYTTCGAT	Carlaviruses	715	This study	Forward
pGV3t	TGGNCNTGCTACCACAANGG	Allexiviruses	950	Chen et al, 2004	Forward
U341	CCGGAATTCATGRTITGGTGYATIGAIAAYGG[1]	Potyviruses	600-850	Langeveld et al., 1991	Forward

Table S3 Primer sequence information for PCR labelling of species-specific probes

Primer name	Sequence (5′-3′)	Genus	Product size (bp)
GarVA_F	TCGTCGAAAACTATTGCACCT	Allexi	507
GarVA_R	GAAGTGCCTTGAAATGGCTTA		
GarVB_F	GGGCAAACAGCAGAATAAGC	Allexi	610
GarVB_R	ATAGCTTAGCGGGTCCTTCA		
GarVC_F	GCTGAACTCAAAGACTTAGT	Allexi	509
GarVC_R	TCTTACGTCCTAATTTCA		
GarVX_F	CACCTTGTGGCATGCCTC	Allexi	500
GarVX_R	GCTTTACGTCCTAATTTC		
ShVX_F	GTGAGTGTCTTTGGTCACGT	Allexi	500
ShVX_R	ACTAGAGCCGACCTCCTAGG		
GCLV_F	GGCAAACCAAAGGTTACACT	Carla	572
GCLV_R	GTAAGATACCTAATCGTCCC		
SLV_F	GCTTTCGCAACACAAGAA	Carla	500
SLV_R	TCGTTCCCACTTATACATTA		
LYSV_F	AGCAGGCTTATATGCCAAGG	Poty	550
LYSV_R	AAAACCGTACTCCGGGTAGA		
OYDV_F	TCAGGGAATTGGACTATGATG	Poty	535
OYDV_R	TCAGATTAAACGGAAATGAAGA		
SYSV_F	TTGGACGATGATGGATGGCG	Poty	500
SYSV_R	CTATGCCACAAACGGTGGTT		
IYSV_F	GTTTAGAAGACTCACCAATG	Tospo	837
IYSV R	ACCAGAGGAAGCCCGCAG		

Table S4 Primer sequence information for amplicon sequencing of the coat protein (CP) of nine garlic virus species

Primer name	Sequence (5′-3′)	Genus	Product size (bp)
GVA_CP_F	ACTGGAAGGGTGAATTAGATGAGT	Allexi	854
GVA_CP_R	TGGCAAARTGYAGGCAGCAA	Allexi	
GVB_CP_F	TCAGGGATGATGGTTTGTGTT	Allexi	878
GVB_CP_R	GGGAGRTTGGGTTGAGCAAA	Allexi	
GVC_CP_F	TCGTGAATGCYGCCATGACT	Allexi	916
GVC_CP_R1	GTGCCCTGGAAGGGCTTATT	Allexi	
GVC_CP_R2	GTGAAATGAAGGCAGCAGAG	Allexi	772
GVX_CP_F	TGCAACCATTCAGCGACTCT	Allexi	910
GVX_CP_R	TGAAGACAGGCGAGCARGTT	Allexi	
GCLV_CP_F	ACGCTTGAGGTAGCCAAACA	Carla	1073
GCLV_CP_R	TAGGCACGCCACTAYCACAC	Carla	
SLV_CP_F	ACAGCGCTCTAAATTGATATTATGACT	Carla	935
SLV_CP_R	ACGGTAAGTTCTYTGYTTGATCA	Carla	
LYSV_CP_F	GATGCACCTCCGGATGAAA	Poty	938
LYSV_CP_R	TTTAAGTGGAAGTCYATAACCGRT	Poty	
OYDV_CP_ F	GCGGGGGAAGGAGAAGATGCAG	Poty	730
OYDV_CP_R	CCGCAGCTGTGTGTCTTTCCG	Poty	
SYSV_CP_F	AATCCTGGATGATCGTATACCG	Poty	923
SYSV_CP_R	ACTATGCCACAAACGYTGGT	Poty	

Table S5 Distance matrix showing percent nucleotide identities of coat protein sequences of potyviruses. Australian isolates from this study (Eastern Australia) are shown in orange and from Western Australia in light blue (GenBank accessions).

4	Y11826	**MH686302**	**MH686301**	**MH686300**	KY347900	KR025485	KF862691	KF632715	KF632714	KF623541	KF623540	JX433020	JX433019	JX429964	JN127342	HQ918256	HQ873798	HQ258894	GQ475410	FJ765739	DQ925455	AY390253	AJ292224	KF597285	JX429967	JN127340	GQ475418	DQ402056	DQ299382	DQ925458	AB693978	AB669435
Y11826 OYDV		82.13	82.04	81.97	96.29	98.07	82.64	81.45	82.94	87.63	95.19	98.98	96.06	81.67	82.25	84.31	82.82	82.28	82.07	82.96	82.49	83.98	82.67	62.54	61.79	62.28	61.9	63.79	59.88	74.37	74.45	74.23
**MH686302 OYDV**	82.13		86.9	99.7	81.53	81.38	85.44	86.34	86.49	81.56	82.13	81.98	81.98	86.41	98.8	81.61	86.96	87.24	86.81	80.06	93.39	81.03	80.06	63.1	62.52	63.17	63.27	63.32	60.16	72.13	71.85	72.15
**MH686301 OYDV**	82.04	86.9		86.6	81.58	81.43	86.91	86.3	87.22	80.4	81.89	82.04	81.28	98.25	87.52	84.13	86.47	99.7	85.71	84.04	86.61	85.3	84.65	62.16	62.31	62.52	62.78	62.39	60.34	75.38	73.8	74.78
**MH686300 OYDV**	81.97	99.7	86.6		81.36	81.21	85.76	86.67	86.52	81.39	81.97	81.82	81.82	86.14	98.79	81.52	86.99	86.82	86.84	79.88	93.33	80.85	79.88	62.78	62.19	62.84	62.94	63.29	60.16	72.33	72.35	72.35
KY347900 OYDV	96.29	81.53	81.58	81.36		95.85	81.6	81.16	81.31	87.56	94.66	96.44	94.96	81.01	81.75	82.66	82.37	81.75	81.63	82.07	82.64	83.36	82.22	62.37	61.79	62.28	62.1	63.89	60.34	73.92	74.45	73.94
R025485 OYDV	98.07	81.38	81.43	81.21	95.85		82.94	81.16	82.05	87.26	95.25	98.22	95.25	80.86	81.31	83.26	82.52	81.6	81.78	82.37	81.75	83.67	82.07	62.23	62.08	61.98	61.96	63.89	60.34	74.37	74.74	74.09
KF862691 OYDV	82.64	85.44	86.91	85.76	81.6	82.94		91.1	86.94	81.93	82.79	82.34	81.6	86.28	85.61	81.91	90.96	87.24	90.22	82.52	85.31	83.05	83.26	62.81	62.23	63.17	62.25	64.6	60.95	74.37	73.42	73.5
KF632715 OYDV	81.45	86.34	86.3	86.67	81.16	81.16	91.1		85.76	80.59	81.6	81.45	80.71	85.98	86.2	81.31	97.48	86.8	96.74	83.11	85.46	83.67	83.56	62.37	60.76	62.13	61.1	61.15	60.34	73.92	73.86	72.91
KF632714 OYDV	82.94	86.49	87.22	86.52	81.31	82.05	86.94	85.76		81.2	80.91	82.14	81.55	86.61	87.28	86.56	85.93	87.43	85.19	82.67	85.46	83.83	82.96	64.91	62.08	63.17	63.3	64.35	60.51	73.32	73.42	74.38
KF623541 OYDV	87.63	81.56	80.4	81.39	87.56	87.26	81.93	80.59	81.2		88.57	87.86	87.43	80.61	81.68	81.93	81.66	80.97	80.92	80.33	81.63	81.52	80.62	62.1	60.91	62.43	61.34	61.94	58.51	72.43	73.17	73.21
KF623540 OYDV	95.19	82.13	81.89	81.97	94.66	95.25	82.79	81.6	80.91	88.57		95.72	96.58	81.61	82.1	82.36	82.67	82.13	81.93	82.22	82.05	83.52	82.22	62.39	62.23	62.13	62.14	63.57	58.73	73.77	73.42	73.06
JX433020 OYDV	98.98	81.98	82.04	81.82	96.44	98.22	82.34	81.45	82.14	87.86	95.72		96.53	81.75	82.1	83.56	82.82	82.28	82.07	82.37	82.34	83.67	82.37	62.25	61.64	62.28	61.67	63.48	59.73	74.37	73.86	74.23
JX433019 OYDV	96.06	81.98	81.28	81.82	94.96	95.25	81.6	80.71	81.55	87.43	96.58	96.53		80.88	81.95	82.81	81.78	81.39	81.04	81.63	82.34	83.36	81.93	62.39	62.23	61.54	61.43	62.67	58.46	73.47	72.98	73.5
JX429964 OYDV	81.67	86.41	98.25	86.14	81.01	80.86	86.28	85.98	86.61	80.61	81.61	81.75	80.88		87.06	83.78	86	98.15	85.56	83.41	85.68	84.45	84	61.95	61.64	62.5	62.7	62.61	60.09	74.67	73.42	74.52
JN127342 OYDV	82.25	98.8	87.52	98.79	81.75	81.31	85.61	86.2	87.28	81.68	82.1	82.1	81.95	87.06		81.91	86.96	87.87	86.52	80.59	93.18	81.49	80.89	63.45	62.81	63.17	63.65	63.27	59.63	71.98	71.95	71.89
HQ918256 OYDV	84.31	81.61	84.13	81.52	82.66	83.26	81.91	81.31	86.56	81.93	82.36	83.56	82.81	83.78	81.91		81.76	84.16	81.91	83.26	81.91	85.15	83.26	62.96	60.62	62.28	62.34	63.99	61.49	73.32	72.85	73.44
HQ873798 OYDV	82.82	86.96	86.47	86.99	82.37	82.52	90.96	97.48	85.93	81.66	82.67	82.82	81.78	86	86.96	81.76		86.96	99.26	83.11	86.67	83.67	83.41	62.37	60.76	62.43	61.24	62.16	60.95	74.52	74.6	73.65
HQ258894 OYDV	82.28	87.24	99.7	86.82	81.75	81.6	87.24	86.8	87.43	80.97	82.13	82.28	81.39	98.15	87.87	84.16	86.96		86.22	84.15	86.35	85.38	84.74	62.48	62.37	62.87	62.98	62.75	60.55	74.96	73.72	74.38
GQ475410 OYDV	82.07	86.81	85.71	86.84	81.63	81.78	90.22	96.74	85.19	80.92	81.93	82.07	81.04	85.56	86.52	81.91	99.26	86.22		82.37	86.22	83.2	82.67	62.23	60.62	62.28	61.1	61.58	60.95	74.67	74.45	73.5
FJ765739 OYDV	82.96	80.06	84.04	79.88	82.07	82.37	82.52	83.11	82.67	80.33	82.22	82.37	81.63	83.41	80.59	83.26	83.11	84.15	82.37		80.74	97.51	97.63	61.64	61.2	61.98	60.81	62.88	59.72	73.77	72.1	73.61
DQ925455 OYDV	82.49	93.39	86.61	93.33	82.64	81.75	85.31	85.46	85.46	81.63	82.05	82.34	82.34	85.68	93.18	81.91	86.67	86.35	86.22	80.74		81.8	80.59	62.52	62.37	62.87	62.97	63.02	60.18	72.28	72.54	73.35
AY390253 OYDV	83.98	81.03	85.3	80.85	83.36	83.67	83.05	83.67	83.83	81.52	83.52	83.67	83.36	84.45	81.49	85.15	83.67	85.38	83.2	97.51	81.8		97.36	63.05	62.6	63.36	62.14	64.79	61.73	75	74.38	75.62
AJ292224 OYDV	82.67	80.06	84.65	79.88	82.22	82.07	83.26	83.56	82.96	80.62	82.22	82.37	81.93	84	80.89	83.26	83.41	84.74	82.67	97.63	80.59	97.36		62.67	62.37	61.98	61.82	62.73	59.57	74.07	72.54	74.34
KF597285 LYSV	62.54	63.1	62.16	62.78	62.37	62.23	62.81	62.37	64.91	62.1	62.39	62.25	62.39	61.95	63.45	62.96	62.37	62.48	62.23	61.64	62.52	63.05	62.67		89.91	82.01	92.63	79.09	80.79	61.79	61.02	62.28
JX429967 LYSV	61.79	62.52	62.31	62.19	61.79	62.08	62.23	60.76	62.08	60.91	62.23	61.64	62.23	61.64	62.81	60.62	60.76	62.37	60.62	61.2	62.37	62.6	62.37	89.91		80.81	93.78	79.88	81.42	62.37	60.88	61.99

JN127340 LYSV WA	62.28	63.17	62.52	62.84	62.28	61.98	63.17	62.13	63.17	62.43	62.13	62.28	61.54	62.5	63.17	62.28	62.43	62.87	62.28	61.98	62.87	63.36	61.98	82.01	80.81		81.14	79.67	82.8	61.69	62.48	62.13
GQ475418 LYSV	61.9	63.27	62.78	62.94	62.1	61.96	62.25	61.1	63.3	61.34	62.14	61.67	61.43	62.7	63.65	62.34	61.24	62.98	61.1	60.81	62.97	62.14	61.82	92.63	93.78	81.14		79.49	79.16	62.81	61.24	62.16
DQ402056 LYSV	63.79	63.32	62.39	63.29	63.89	63.89	64.6	61.15	64.35	61.94	63.57	63.48	62.67	62.61	63.27	63.99	62.16	62.75	61.58	62.88	63.02	64.79	62.73	79.09	79.88	79.67	79.49		82.1	60.97	61.53	60.63
DQ299382 LYSV	59.88	60.16	60.34	60.16	60.34	60.34	60.95	60.34	60.51	58.51	58.73	59.73	58.46	60.09	59.63	61.49	60.95	60.55	60.95	59.72	60.18	61.73	59.57	80.79	81.42	82.8	79.16	82.1		60.13	60.64	60.86
DQ925458 SYSV	74.37	72.13	75.38	72.33	73.92	74.37	74.37	73.92	73.32	72.43	73.77	74.37	73.47	74.67	71.98	73.32	74.52	74.96	74.67	73.77	72.28	75	74.07	61.79	62.37	61.69	62.81	60.97	60.13		90.05	90.18
AB693978 SYSV	74.45	71.85	73.8	72.35	74.45	74.74	73.42	73.86	73.42	73.17	73.42	73.86	72.98	73.42	71.95	72.85	74.6	73.72	74.45	72.1	72.54	74.38	72.54	61.02	60.88	62.48	61.24	61.53	60.64	90.05		88.87
AB669435 SYSV	74.23	72.15	74.78	72.35	73.94	74.09	73.5	72.91	74.38	73.21	73.06	74.23	73.5	74.52	71.89	73.44	73.65	74.38	73.5	73.61	73.35	75.62	74.34	62.28	61.99	62.13	62.16	60.63	60.86	90.18	88.87	

Table S6 Distance matrix showing percent nucleotide identities of coat protein sequences of carlaviruses. Australian isolates from this study (Eastern Australia) are shown in orange and from Western Australia in light blue (GenBank accessions).

	**MH686306**	**MH686305**	LC279526	JX429968	JX429966	JQ899443	JF320811	HQ258896	GU355922	GU120175	DQ520093	AJ409316	AB004802	AB004686	AB004686	AB004567	KP208802	KJ801307	KF862699	JQ899445	JQ818259	JF320810	HQ873863	HQ873861	**MH686304**	**MH686303**	DQ520092	AF228416	AB004804	AB004566
**MH686306 SLV**		92.58	78.11	89.22	91.44	86.47	89.21	92.34	97.48	88.62	89.33	92.58	87.65	94.01	94.01	89.09	55.75	54.82	55.05	54.36	55.05	54.01	54.94	54.82	55.52	55.75	55.17	54.82	54.01	55.75
**MH686305 SLV**	92.58		78.20	89.93	96.52	85.95	89.10	98.83	92.73	88.76	89.45	98.94	88.04	92.85	92.85	89.22	55.73	55.52	55.06	54.49	55.06	54.73	54.72	54.61	55.29	55.52	55.29	55.29	54.72	55.75
LC279526 SLV	78.11	78.20		79.39	79.87	78.51	78.92	78.10	78.92	78.83	79.63	78.31	80.56	79.51	79.51	79.39	54.02	54.28	53.62	52.85	53.84	53.46	52.96	52.85	53.51	53.51	54.06	54.28	53.51	53.29
JX429968 SLV	89.22	89.93	79.39		89.49	88.19	90.07	89.97	90.04	88.29	89.10	90.28	88.98	89.92	89.92	89.57	56.38	55.70	55.48	54.61	55.15	55.51	54.72	54.61	55.81	56.03	55.92	55.81	55.15	54.93
JX429966 SLV	91.44	96.52	79.87	89.49		87.89	92.17	96.81	91.61	89.95	91.10	96.65	89.81	91.87	91.87	90.71	56.97	55.96	55.46	54.61	54.84	54.41	55.21	54.96	54.89	55.00	55.46	55.58	54.81	55.54
JQ899443 SLV	86.47	85.95	78.51	88.19	87.89		90.16	86.32	87.10	89.46	89.80	86.89	89.33	86.17	86.17	90.39	56.76	56.36	56.59	55.57	55.91	56.21	55.11	55.00	56.02	56.25	56.71	55.34	55.46	55.57
JF320811 SLV	89.21	89.10	78.92	90.07	92.17	90.16		89.50	89.67	92.03	93.90	89.45	91.78	89.32	89.32	94.37	56.27	55.81	55.59	55.04	54.72	55.73	54.93	54.72	55.81	56.03	55.92	55.04	55.15	55.59
HQ258896 SLV	92.34	98.83	78.10	89.97	96.81	86.32	89.50		92.73	89.34	89.92	99.06	88.63	92.97	92.97	89.68	55.56	54.46	53.92	53.00	54.14	53.78	53.71	53.60	54.08	54.18	54.35	54.46	53.28	54.02
GU355922 GLV	97.48	92.73	78.92	90.04	91.61	87.10	89.67	92.73		88.75	89.32	92.97	88.03	94.25	94.25	89.32	55.49	54.84	55.06	54.04	55.18	54.28	55.06	54.95	55.52	55.75	55.29	54.61	54.15	55.86
GU120175 SLV	88.62	88.76	78.83	88.29	89.95	89.46	92.03	89.34	88.75		92.15	89.34	91.33	88.28	88.28	92.26	56.22	55.97	55.29	55.40	55.86	55.19	54.04	53.93	55.75	55.97	55.75	55.06	54.84	56.43
DQ520093 GLV	89.33	89.45	79.63	89.10	91.10	89.80	93.90	89.92	89.32	92.15		90.15	90.85	89.32	89.32	98.12	56.46	56.31	56.77	55.29	55.75	55.64	55.52	55.40	56.66	56.88	56.54	55.75	55.29	55.75
AJ409316 GLV	92.58	98.94	78.31	90.28	96.65	86.89	89.45	99.06	92.97	89.34	90.15		88.28	93.08	93.08	89.92	55.98	55.40	55.40	54.61	55.18	54.85	54.95	54.84	55.18	55.40	55.18	55.18	54.84	55.86
AB004802 SLV)	87.65	88.04	80.56	88.98	89.81	89.33	91.78	88.63	88.03	91.33	90.85	88.28		87.91	87.91	91.43	56.71	56.88	56.09	56.31	55.86	56.21	55.18	55.06	56.77	57.00	56.77	55.97	56.31	55.63
AB004686 GLV	94.01	92.85	79.51	89.92	91.87	86.17	89.32	92.97	94.25	88.28	89.32	93.08	87.91		100.00	89.09	55.61	54.61	54.72	53.58	54.61	53.82	54.49	54.38	54.72	54.95	54.49	54.04	54.49	55.18
AB004686 GLV	94.01	92.85	79.51	89.92	91.87	86.17	89.32	92.97	94.25	88.28	89.32	93.08	87.91	100.00		89.09	55.61	54.61	54.72	53.58	54.61	53.82	54.49	54.38	54.72	54.95	54.49	54.04	54.49	55.18
AB004567 GLV	89.09	89.22	79.39	89.57	90.71	90.39	94.37	89.68	89.32	92.26	98.12	89.92	91.43	89.09	89.09		56.83	55.86	56.54	55.40	55.52	55.30	55.06	54.95	55.97	56.20	56.20	55.29	55.29	55.86
KP208802 GCLV	55.75	55.73	54.02	56.38	56.97	56.76	56.27	55.56	55.49	56.22	56.46	55.98	56.71	55.61	55.61	56.83		90.45	93.27	88.77	89.26	89.02	93.28	93.40	89.47	89.72	89.99	89.36	90.45	90.09
KJ801307 GCLV	54.82	55.52	54.28	55.70	55.96	56.36	55.81	54.46	54.84	55.97	56.31	55.40	56.88	54.61	54.61	55.86	90.45		89.07	93.46	95.85	95.35	89.29	89.40	96.57	96.88	96.57	95.32	94.70	89.81
KF862699 GCLV	55.05	55.06	53.62	55.48	55.46	56.59	55.59	53.92	55.06	55.29	56.77	55.40	56.09	54.72	54.72	56.54	93.27	89.07		87.94	87.73	87.79	91.88	91.88	88.24	88.45	88.15	88.03	89.29	87.62
JQ899445 GCLV	54.36	54.49	52.85	54.61	54.61	55.57	55.04	53.00	54.04	55.40	55.29	54.61	56.31	53.58	53.58	55.40	88.77	93.46	87.94		92.52	92.82	88.06	88.16	92.47	92.12	93.45	92.63	93.90	88.53
JQ818259 GCLV	55.05	55.06	53.84	55.15	54.84	55.91	54.72	54.14	55.18	55.86	55.75	55.18	55.86	54.61	54.61	55.52	89.26	95.85	87.73	92.52		93.95	88.45	88.55	95.33	95.64	96.35	94.17	93.36	89.41
JF320810 GCLV	54.01	54.73	53.46	55.51	54.41	56.21	55.73	53.78	54.28	55.19	55.64	54.85	56.21	53.82	53.82	55.30	89.02	95.35	87.79	92.82	93.95		88.27	88.39	93.92	94.05	95.00	93.50	93.83	90.51
HQ873863 GCLV	54.94	54.72	52.96	54.72	55.21	55.11	54.93	53.71	55.06	54.04	55.52	54.95	55.18	54.49	54.49	55.06	93.28	89.29	91.88	88.06	88.45	88.27		99.58	88.05	88.36	88.66	88.65	89.10	88.88
HQ873861 GCLV	54.82	54.61	52.85	54.61	54.96	55.00	54.72	53.60	54.95	53.93	55.40	54.84	55.06	54.38	54.38	54.95	93.40	89.40	91.88	88.16	88.55	88.39	99.58		88.15	88.46	88.76	88.75	89.20	88.88
**MH686304 GCLV**	55.52	55.29	53.51	55.81	54.89	56.02	55.81	54.08	55.52	55.75	56.66	55.18	56.77	54.72	54.72	55.97	89.47	96.57	88.24	92.47	95.33	93.92	88.05	88.15		99.30	96.47	94.80	93.97	88.93
**MH686303 GCLV**	55.75	55.52	53.51	56.03	55.00	56.25	56.03	54.18	55.75	55.97	56.88	55.40	57.00	54.95	54.95	56.20	89.72	96.88	88.45	92.12	95.64	94.05	88.36	88.46	99.30		96.78	94.91	93.68	88.50
DQ520092 GCLV	55.17	55.29	54.06	55.92	55.46	56.71	55.92	54.35	55.29	55.75	56.54	55.18	56.77	54.49	54.49	56.20	89.99	96.57	88.15	93.45	96.35	95.00	88.66	88.76	96.47	96.78		95.32	94.09	89.20
AF228416 GCLV	54.82	55.29	54.28	55.81	55.58	55.34	55.04	54.46	54.61	55.06	55.75	55.18	55.97	54.04	54.04	55.29	89.36	95.32	88.03	92.63	94.17	93.50	88.65	88.75	94.80	94.91	95.32		93.15	88.57
AB004804 GCLV	54.01	54.72	53.51	55.15	54.81	55.46	55.15	53.28	54.15	54.84	55.29	54.84	56.31	54.49	54.49	55.29	90.45	94.70	89.29	93.90	93.36	93.83	89.10	89.20	93.97	93.68	94.09	93.15		90.47
AB004566 GCLV	55.75	55.75	53.29	54.93	55.54	55.57	55.59	54.02	55.86	56.43	55.75	55.86	55.63	55.18	55.18	55.86	90.09	89.81	87.62	88.53	89.41	90.51	88.88	88.88	88.93	88.50	89.20	88.57	90.47	

Table S7 Distance matrix showing percent nucleotide identities of coat protein sequences of allexiviruses. Australian isolates from this study (Eastern Australia) are shown in orange and from Western Australia in light blue (GenBank accessions).

	KU933691	JX997952	JX997951	JX488633	JX429970	JQ899446	JQ899444	JN019812	**MH686308**	**MH686307**	AF478197	AB010300	KP657933	KM379144	JX488620	JN019813	**MH686310**	**MH686309**	AF543829	AB010301	KX034780	KF955566	JQ899447	JN019814	HM777004	KF555653	KF446210	JX488630	JN019815	LN875277	KP713693	KF530328	JX429971	JX429969	JQ807994	GQ475426	**MH686313**	**H686312**	**MH686311**	AJ292229
KU933691 GarVA		83.99	78.06	97.49	85.90	97.35	74.21	85.24	97.62	97.82	97.22	97.52	61.62	62.34	61.49	62.60	61.71	61.81	61.42	61.81	60.58	59.26	62.11	62.37	59.10	71.92	71.26	68.77	68.28	64.94	64.28	64.04	64.17	65.22	63.52	64.54	63.52	63.78	64.83	62.99
JX997952 GarVA	83.99		78.95	83.47	98.61	83.40	75.65	83.27	83.29	83.20	82.49	83.04	61.85	62.61	62.25	62.48	61.66	61.06	61.19	62.48	62.06	62.06	61.53	62.18	62.30	71.96	72.16	70.21	70.29	65.83	65.17	64.55	64.04	64.55	64.04	64.77	64.17	64.20	65.11	64.42
JX997951 GarVA	78.06	78.95		78.98	80.37	78.90	73.10	77.98	79.29	79.10	78.51	78.67	58.96	60.47	59.23	60.21	58.66	58.12	58.25	59.95	60.60	60.08	60.47	61.13	59.92	69.14	69.71	67.63	67.06	62.95	63.61	62.70	62.57	62.44	62.44	62.28	62.04	62.04	62.04	61.91
JX488633 GarVA	97.49	83.47	78.98		85.19	98.68	74.08	84.98	99.07	99.27	98.68	98.84	61.49	62.47	61.49	62.47	61.57	61.68	61.16	61.68	60.97	59.53	62.11	62.37	59.37	71.65	71.00	69.16	68.68	65.21	64.28	64.30	64.17	65.22	63.78	64.28	63.78	64.04	65.09	62.99
JX429970 GarVA	85.90	98.61	80.37	85.19		84.88	77.99	83.56	84.91	85.03	84.18	84.86	63.31	61.95	63.87	62.95	63.32	62.42	62.15	62.60	62.69	62.97	63.15	64.15	63.24	74.45	74.30	71.35	71.53	67.37	66.67	66.44	65.23	66.30	65.70	66.11	65.88	65.97	67.09	66.30
JQ899446 GarVA	97.35	83.40	78.90	98.68	84.88		72.78	85.21	98.83	98.80	98.24	98.38	61.62	60.78	61.49	60.78	61.66	60.42	59.80	59.93	61.14	59.11	60.97	61.24	58.96	70.98	71.52	69.42	68.85	65.21	64.54	63.30	62.38	63.68	62.64	64.54	62.67	63.10	64.11	61.92
JQ899444 GarVA	74.21	75.65	73.10	74.08	77.99	72.78		71.59	73.28	73.56	73.43	73.86	59.92	59.17	59.79	59.29	59.26	57.82	58.31	57.70	58.45	58.83	60.25	60.03	59.31	67.63	68.41	67.54	66.84	62.58	63.92	61.18	60.32	61.45	61.15	62.45	60.55	60.84	60.96	60.68
JN019812 GarVA	85.24	83.27	77.98	84.98	83.56	85.21	71.59		85.07	84.59	84.44	84.74	60.03	60.80	60.56	60.80	59.83	59.18	60.18	58.80	61.89	61.51	59.88	60.15	61.36	69.96	70.85	69.82	69.76	64.41	63.35	62.80	62.30	61.81	62.89	63.08	63.05	63.23	63.36	62.30
**MH686308 GarVA**	97.62	83.29	79.29	99.07	84.91	98.83	73.28	85.07		99.41	98.83	98.99	61.36	61.84	61.22	61.84	61.44	61.06	60.41	60.93	60.36	58.81	61.61	61.74	58.65	70.38	70.74	68.90	68.33	65.07	64.68	64.04	63.78	64.81	63.39	64.54	63.39	63.39	64.42	62.61
**MH686307 GarVA**	97.82	83.20	79.10	99.27	85.03	98.80	73.56	84.59	99.41		99.05	99.46	61.62	61.65	61.22	61.65	61.66	60.90	60.40	60.78	60.56	58.90	60.87	61.24	58.74	70.67	71.06	69.23	68.65	64.94	64.54	63.38	63.03	64.15	62.85	64.54	62.88	63.31	64.08	62.12
AF478197 GarVA	97.22	82.49	78.51	98.68	84.18	98.24	73.43	84.44	98.83	99.05		98.89	61.36	61.13	61.22	61.13	61.40	60.63	60.13	60.50	60.56	58.90	60.65	60.93	58.74	70.48	70.87	69.29	68.72	64.41	64.01	62.88	63.00	63.90	62.59	64.54	62.37	62.80	63.57	61.62
AB010300 GarVA	97.52	83.04	78.67	98.84	84.86	98.38	73.86	84.74	98.99	99.46	98.89		61.62	61.97	61.22	61.97	61.80	60.96	60.33	61.08	60.36	59.07	60.73	60.99	58.92	70.56	71.03	69.29	68.72	65.07	64.54	63.20	63.22	64.22	62.66	64.68	62.69	63.12	64.15	62.18
KP657933 GarVB	61.62	61.85	58.96	61.49	63.31	61.62	59.92	60.03	61.36	61.62	61.36	61.62		89.67	97.96	89.00	98.23	97.69	94.83	89.30	65.95	66.76	66.80	66.40	66.62	62.17	61.77	61.73	62.45	77.21	75.85	76.26	74.22	75.31	76.12	75.85	75.31	75.58	76.93	75.31
KM379144 GarVB	62.34	62.61	60.47	62.47	61.95	60.78	59.17	60.80	61.84	61.65	61.13	61.97	89.67		90.22	97.92	89.68	88.65	89.88	90.74	64.05	63.54	64.74	64.53	64.04	60.70	61.96	62.71	63.34	77.75	77.34	75.66	74.11	75.75	75.60	76.80	74.78	75.45	76.72	75.91
JX488620 GarVB	61.49	62.25	59.23	61.49	63.87	61.49	59.79	60.56	61.22	61.22	61.22	61.22	97.96	90.22		89.54	98.10	97.69	95.92	90.38	66.22	66.89	66.53	66.40	66.76	62.57	62.43	62.40	63.25	77.21	76.26	76.93	74.90	75.85	77.07	76.26	75.98	76.26	77.21	75.58
JN019813 GarVB	62.60	62.48	60.21	62.47	62.95	60.78	59.29	60.80	61.84	61.65	61.13	61.97	89.00	97.92	89.54		89.01	88.03	88.90	90.24	64.18	63.92	64.78	64.82	64.42	60.08	61.57	62.06	62.68	77.88	77.48	75.73	73.92	75.82	75.66	76.93	74.84	75.51	76.79	75.98
**MH686310 GarVB**	61.71	61.66	58.66	61.57	63.32	61.66	59.26	59.83	61.44	61.66	61.40	61.80	98.23	89.68	98.10	89.01		98.12	94.63	89.84	65.29	66.22	65.59	65.46	65.95	61.82	61.73	61.69	62.27	76.66	76.12	75.64	73.49	74.83	75.50	76.26	74.70	75.37	76.04	74.57
**MH686309 GarVB**	61.81	61.06	58.12	61.68	62.42	60.42	57.82	59.18	61.06	60.90	60.63	60.96	97.69	88.65	97.69	88.03	98.12		94.13	89.04	64.43	64.68	64.29	64.32	64.42	60.33	61.57	61.40	62.16	76.93	76.26	75.22	71.57	74.31	75.41	76.66	74.34	75.00	75.64	74.08
AF543829 GarVB	61.42	61.19	58.25	61.16	62.15	59.80	58.31	60.18	60.41	60.40	60.13	60.33	94.83	89.88	95.92	88.90	94.63	94.13		89.90	65.19	65.06	64.66	65.06	65.44	60.08	61.57	61.00	61.63	76.39	75.71	74.12	71.61	73.33	74.06	75.44	73.23	73.89	74.90	73.11
AB010301 GarVB	61.81	62.48	59.95	61.68	62.60	59.93	57.70	58.80	60.93	60.78	60.50	61.08	89.30	90.74	90.38	90.24	89.84	89.04	89.90		63.04	63.29	62.56	62.22	63.91	60.58	61.96	61.53	62.16	76.32	75.37	74.53	72.38	74.37	74.59	76.18	73.90	74.56	74.94	74.15
KX034780 GarVC	60.58	62.06	60.60	60.97	62.69	61.14	58.45	61.89	60.36	60.56	60.56	60.36	65.95	64.05	66.22	64.18	65.29	64.43	65.19	63.04		92.18	75.61	75.13	91.25	59.77	61.57	62.06	62.50	66.80	66.53	64.35	64.22	64.73	64.98	67.07	64.35	64.60	65.23	63.97
KF955566 GarVC	59.26	62.06	60.08	59.53	62.97	59.11	58.83	61.51	58.81	58.90	58.90	59.07	66.76	63.54	66.89	63.92	66.22	64.68	65.06	63.29	92.18		75.35	75.38	98.07	61.05	62.75	62.19	62.50	66.13	66.53	64.22	63.84	64.22	64.48	65.86	64.22	64.48	64.60	63.72
JQ899447 GarVC	62.11	61.53	60.47	62.11	63.15	60.97	60.25	59.88	61.61	60.87	60.65	60.73	66.80	64.74	66.53	64.78	65.59	64.29	64.66	62.56	75.61	75.35		96.03	75.39	60.40	62.44	61.27	60.79	67.51	67.91	64.49	63.98	64.87	63.84	66.58	63.50	63.93	65.55	64.86
JN019814 GarVC	62.37	62.18	61.13	62.37	64.15	61.24	60.03	60.15	61.74	61.24	60.93	60.99	66.40	64.53	66.40	64.82	65.46	64.32	65.06	62.22	75.13	75.38	96.03		75.42	60.53	62.57	61.27	60.79	67.38	68.18	64.58	63.86	64.83	63.80	66.85	63.47	64.02	65.26	64.82
HM777004 GarVC	59.10	62.30	59.92	59.37	63.24	58.96	59.31	61.36	58.65	58.74	58.74	58.92	66.62	64.04	66.76	64.42	65.95	64.42	65.44	63.91	91.25	98.07	75.39	75.42		60.77	62.34	61.77	62.48	66.13	66.53	63.71	63.96	64.09	64.21	66.13	63.83	64.09	64.72	63.83
KF555653 GarVD	71.92	71.96	69.14	71.65	74.45	70.98	67.63	69.96	70.38	70.67	70.48	70.56	62.17	60.70	62.57	60.08	61.82	60.33	60.08	60.58	59.77	61.05	60.40	60.53	60.77		97.48	87.88	87.12	62.40	62.01	60.50	60.80	60.53	61.23	61.87	60.63	61.04	61.04	60.50
KF446210 GarVD	71.26	72.16	69.71	71.00	74.30	71.52	68.41	70.85	70.74	71.06	70.87	71.03	61.77	61.96	62.43	61.57	61.73	61.57	61.57	61.96	61.57	62.75	62.44	62.57	62.34	97.48		88.54	87.76	62.40	62.53	61.80	62.45	61.93	62.45	62.14	61.67	61.93	62.06	61.67
JX488630 GarVD	68.77	70.21	67.63	69.16	71.35	69.42	67.54	69.82	68.90	69.23	69.29	69.29	61.73	62.71	62.40	62.06	61.69	61.40	61.00	61.53	62.06	62.19	61.27	61.27	61.77	87.88	88.54		96.95	61.84	61.57	61.37	61.76	61.50	61.50	61.57	61.24	61.50	61.24	60.84
JN019815 GarVD	68.28	70.29	67.06	68.68	71.53	68.85	66.84	69.76	68.33	68.65	68.72	68.72	62.45	63.34	63.25	62.68	62.27	62.16	61.63	62.16	62.50	62.50	60.79	60.79	62.48	87.12	87.76	96.95		62.95	63.08	62.65	62.91	62.78	62.91	62.82	62.52	62.78	62.65	61.99
LN875277 GarVX	64.94	65.83	62.95	65.21	67.37	65.21	62.58	64.41	65.07	64.94	64.41	65.07	77.21	77.75	77.21	77.88	76.66	76.93	76.39	76.32	66.80	66.13	67.51	67.38	66.13	62.40	62.40	61.84	62.95		91.68	95.22	92.36	95.23	94.67	94.54	94.81	94.81	99.32	94.81
KP713693 GarVX	64.28	65.17	63.61	64.28	66.67	64.54	63.92	63.35	64.68	64.54	64.01	64.54	75.85	77.34	76.26	77.48	76.12	76.26	75.71	75.37	66.53	66.53	67.91	68.18	66.53	62.01	62.53	61.57	63.08	91.68		92.50	91.26	93.03	91.27	91.41	91.27	91.27	91.27	91.68
KF530328 GarVX	64.04	64.55	62.70	64.30	66.44	63.30	61.18	62.80	64.04	63.38	62.88	63.20	76.26	75.66	76.93	75.73	75.64	75.22	74.12	74.53	64.35	64.22	64.49	64.58	63.71	60.50	61.80	61.37	62.65	95.22	92.50		92.34	95.49	97.86	95.36	98.08	97.81	95.01	95.05
JX429971 GarVX	64.17	64.04	62.57	64.17	65.23	62.38	60.32	62.30	63.78	63.03	63.00	63.22	74.22	74.11	74.90	73.92	73.49	71.57	71.61	72.38	64.22	63.84	63.98	63.86	63.96	60.80	62.45	61.76	62.91	92.36	91.26	92.34		94.19	92.41	92.50	91.97	91.60	91.81	91.76
JX429969 GarVX	65.22	64.55	62.44	65.22	66.30	63.68	61.45	61.81	64.81	64.15	63.90	64.22	75.31	75.75	75.85	75.82	74.83	74.31	73.33	74.37	64.73	64.22	64.87	64.83	64.09	60.53	61.93	61.50	62.78	95.23	93.03	95.49	94.19		95.14	95.09	94.96	94.76	95.21	94.77
JQ807994 GarVX	63.52	64.04	62.44	63.78	65.70	62.64	61.15	62.89	63.39	62.85	62.59	62.66	76.12	75.60	77.07	75.66	75.50	75.41	74.06	74.59	64.98	64.48	63.84	63.80	64.21	61.23	62.45	61.50	62.91	94.67	91.27	97.86	92.41	95.14		94.95	98.32	98.05	94.60	94.41
GQ475426 GarVX	64.54	64.77	62.28	64.28	66.11	64.54	62.45	63.08	64.54	64.54	64.54	64.68	75.85	76.80	76.26	76.93	76.26	76.66	75.44	76.18	67.07	65.86	66.58	66.85	66.13	61.87	62.14	61.57	62.82	94.54	91.41	95.36	92.50	95.09	94.95		94.40	94.67	94.13	93.99
**MH686313 GarVX**	63.52	64.17	62.04	63.78	65.88	62.67	60.55	63.05	63.39	62.88	62.37	62.69	75.31	74.78	75.98	74.84	74.70	74.34	73.23	73.90	64.35	64.22	63.50	63.47	63.83	60.63	61.67	61.24	62.52	94.81	91.27	98.08	91.97	94.96	98.32	94.40		99.51	94.67	94.60
**MH686312 GarVX**	63.78	64.20	62.04	64.04	65.97	63.10	60.84	63.23	63.39	63.31	62.80	63.12	75.58	75.45	76.26	75.51	75.37	75.00	73.89	74.56	64.60	64.48	63.93	64.02	64.09	61.04	61.93	61.50	62.78	94.81	91.27	97.81	91.60	94.76	98.05	94.67	99.51		94.51	94.27

**MH686311 GarVX**	64.83	65.11	62.04	65.09	67.09	64.11	60.96	63.36	64.42	64.08	63.57	64.15	76.93	76.72	77.21	76.79	76.04	75.64	74.90	74.94	65.23	64.60	65.55	65.26	64.72	61.04	62.06	61.24	62.65	99.32	91.27	95.01	91.81	95.21	94.60	94.13	94.67	94.51		94.65
AJ292229 GarVX	62.99	64.42	61.91	62.99	66.30	61.92	60.68	62.30	62.61	62.12	61.62	62.18	75.31	75.91	75.58	75.98	74.57	74.08	73.11	74.15	63.97	63.72	64.86	64.82	63.83	60.50	61.67	60.84	61.99	94.81	91.68	95.05	91.76	94.77	94.41	93.99	94.60	94.27	94.65	
